# Factors associated with morbidities among persons with disabilities in Malaysia

**DOI:** 10.1038/s41598-025-24047-y

**Published:** 2026-01-21

**Authors:** Siti Hafizah Zulkiply, Mohamad Aznuddin Abd Razak, Nor’Ain Ab Wahab, Norliza Shamsudin, Kim Sui Wan, Kishwen Kanna Yoga Ratnam, Yoga Ratnam, Khairul Hasnan Amali

**Affiliations:** Institute for Public Health, National Institute of Health, Ministry of Health, Shah Alam, Selangor Malaysia

**Keywords:** Disability, Washington group, National health and morbidity survey, Population-based survey, Malaysia, Hypertension, Diabetes

## Abstract

Persons with disabilities (PWDs) face disproportionate health risks, particularly from non-communicable diseases (NCDs) such as diabetes and hypertension, which remain leading causes of mortality globally. In lower-middle-income countries (LMICs) like Malaysia, disability and NCDs perpetuate a cycle of vulnerability, yet evidence on morbidity patterns among PWDs remains limited. This study aimed to determine the prevalence and factors associated with morbidity among PWDs in Malaysia. Data were drawn from the National Health and Morbidity Survey (NHMS) 2023: Non-Communicable Diseases, a nationally representative cross-sectional survey of adults aged ≥ 18 years, using a multi-stage stratified sampling design. Disability was assessed with the locally validated Washington Group Short Set, and morbidity was defined as the presence of either diabetes, hypertension or hypercholesterolemia. Multivariate binary logistic regression with complex sample analysis was performed using SPSS v25.0. The prevalence of disability among Malaysian adults was 8.2% (95% CI: 7.37–9.17). Among PWDs, 73.9% reported comorbidities, with hypertension (56.9%) being most prevalent. Compared with older PWDs, those aged 18–35 years (aOR = 0.123; 95% CI: 0.052–0.300) and 36–59 years (aOR = 0.443; 95% CI: 0.247–0.739) had significantly lower odds of morbidity. Education was protective, with secondary (aOR = 0.608; 95% CI: 0.350–1.057) and tertiary education (aOR = 0.270; 95% CI: 0.104–0.586) associated with reduced odds compared with no formal education. Ethnicity also emerged as a determinant, with those categorised as “Others” showing lower morbidity risk than Malays (aOR = 0.360; 95% CI: 0.111–1.166). While the prevalence of disability in Malaysia mirrors international estimates, the burden of morbidity among PWDs is alarmingly high. Targeted strategies, including enhanced screening, health literacy initiatives, and equitable access to care, are urgently needed, particularly for ethnic minorities and individuals with lower educational attainment, to reduce health disparities and improve outcomes for this vulnerable population.

## Introduction

The International Classification of Functioning, Disability and Health (ICF) conceptualises disability as an overarching construct encompassing impairments, activity limitations, and participation restrictions. In parallel, the United Nations Convention on the Rights of Persons with Disabilities underscores the right of all individuals with disabilities to equitable opportunities and fundamental freedoms, highlighting the critical need for reliable statistics to inform policy and practice. According to the World Health Organisation (WHO), the global prevalence of disability is estimated at 16.0%, though reported rates vary widely across countries depending on measurement approaches, ranging from 3.1% to 26.8% ^1,2^.

Non-communicable diseases (NCDs) remain the foremost cause of mortality worldwide, accounting for approximately 63% of deaths annually, with the majority (80%) occurring in low- and middle-income countries (LMICs)^3^. As an LMIC, Malaysia bears a substantial NCD burden, particularly diabetes^4^. Disability and NCDs are intricately linked, often reinforcing one another. Studies have shown that more than one-third (34.2%) of persons with disabilities (PWDs) live with at least one NCD, particularly diabetes and hypertension^5,6^. Sedentary lifestyles, reduced mobility, and socioeconomic disadvantages contribute to elevated NCD risks among PWDs, who are also disproportionately affected by cardiovascular disease^7–9^. Conversely, NCDs, including cardiovascular disease, cancer, and diabetes, can themselves result in disability, either directly (e.g., diabetic retinopathy) or indirectly (e.g., physical limitations associated with cancer)^10^.

Disability thus constitutes a major public health challenge, with far-reaching implications across the life course. Young PWDs frequently encounter barriers in education and employment^11,12^, while older adults experience restricted social participation, increased healthcare utilisation, and poorer quality of life^13–15^. Inequities also extend to healthcare access, as PWDs face challenges in screening, diagnosis, and disease management, contributing to elevated risks of morbidity and premature mortality^16–19^. These disparities are particularly pronounced among women, older adults, and individuals with lower educational attainment.

Addressing the needs of PWDs requires robust policies and forward-looking strategies. Malaysia’s Ministry of Health previously implemented the Plan of Action for Health Care for Persons with Disabilities (2011–2020), which aimed to improve access to services, empower communities, and ensure a competent workforce. However, with Malaysia transitioning into an ageing society, a parallel rise in disability prevalence is anticipated^1,20,21^. Reliable evidence on morbidity patterns and their determinants among PWDs is therefore essential to guide disability-inclusive policies and healthcare planning. In particular, understanding the burden of NCDs in this population is crucial to mitigating adverse outcomes and ensuring equitable access to care.

Existing evidence suggests that sociodemographic factors, including age, sex, education, socioeconomic status, and income, are associated with both NCDs and disability^20–24^. Against this backdrop, the present study seeks to determine the prevalence of morbidity among PWDs in Malaysia and to identify the sociodemographic factors associated with these conditions. Findings from this work are expected to inform the development of targeted prevention strategies and inclusive health policies to improve the health and well-being of PWDs.

## Method

### Study design and sampling

This study represents a secondary analysis of the National Health and Morbidity Survey (NHMS) 2023, a nationally representative cross-sectional survey of adults aged ≥ 18 years residing in Malaysia. A multistage stratified random sampling strategy was employed to ensure representativeness. The Department of Statistics Malaysia constructed the sampling frame, which comprised geographical units known as Enumeration Blocks (EBs).

The sample size was calculated using a single-proportion formula to estimate prevalence. A total of 499 EBs were selected, and from each EB, 12 living quarters (LQs) were randomly sampled. Sample allocation across Malaysia’s 13 states and three federal territories was conducted using probability proportional to size (PPS). Data collection was carried out through face-to-face interviews by trained research assistants using validated questionnaires installed on handheld devices with the REDCap system.

### Study instrument

Data on diabetes and hypercholesterolemia were obtained via structured questionnaires and capillary measurements, while hypertension was assessed using questionnaires and blood pressure measurements. The questionnaire was adapted from the WHO STEPS Instrument. Capillary blood glucose was measured using the Accu-Chek portable system, while hypercholesterolemia was measured using Cardiochek PA 3 in 1, and blood pressure was recorded using the Omron Digital Automatic Blood Pressure Monitor, Model HEM-907.

Disability status was assessed using the Washington Group (WG) Short Set Questionnaire, which covers six functional domains: seeing, hearing, walking, cognition, self-care, and communication. Each item was scored on a four-point scale: (1) no difficulty, (2) some difficulty, (3) a lot of difficulty, and (4) cannot do at all. The instrument has been validated for use in the Malaysian population^25^.

### Variables definition

#### Sociodemographic

Data were collected on sex, age, marital status, ethnicity, education level, household income, and locality. Age was categorised into four groups (18–29, 30–49, 50–59, ≥ 60 years). Ethnicity was classified as Malay, Chinese, Indian, Bumiputera Sabah, Bumiputera Sarawak, or Others. Education was grouped as no formal education, primary, secondary, or tertiary. Marital status was defined as single, married/living with a partner, or divorced/widowed/separated. Household income was categorised into Bottom 40% (B40), Middle 40% (M40), and Top 20% (T20). Locality was defined as urban or rural.

#### Disability

Disability was defined as (1) reporting “a lot of difficulty” or “cannot do at all” in at least one of the six domains, or (2) reporting “some difficulty” in at least two domains^26^.

#### Morbidities

Morbidity was operationally defined as the presence of diabetes, hypertension or hypercholesterolemia. Diabetes was defined as self-reported physician-diagnosed diabetes or raised blood glucose in individuals without known diabetes, using thresholds of fasting blood glucose > 7.0 mmol/L or random blood glucose > 11.1 mmol/L^27^. Hypertension was defined as self-reported physician-diagnosed hypertension or raised blood pressure in individuals without known hypertension. Blood pressure was measured three times, and the mean of the second and third readings was used. Raised blood pressure was defined as an average systolic ≥ 140 mmHg and/or diastolic ≥ 90 mmHg^28^. Hypercholesterolemia was defined as self-reported physician-diagnosed or raised total cholesterol in individuals without known hypercholesterolemia, using a threshold of 5.2mmol/L or more^29^.

### Statistical analysis

All analyses were conducted using IBM SPSS Statistics. Sampling weights were applied to account for the complex survey design, non-response, and differential probabilities of selection. The final weight was derived as *W = W1 × W2 × F × PS*, where W1 is the inverse probability of EB selection, W2 the inverse probability of LQ selection, F a non-response adjustment factor, and PS a post-stratification factor based on age, sex, and ethnicity. Descriptive statistics summarised participant characteristics, and multiple logistic regression was performed to examine factors associated with morbidity among PWDs. Statistical significance was set at *p* < 0.05.

### Ethical approval

Written informed consent was obtained from all participants. The NHMS 2023 protocol received approval from the Medical Research and Ethics Committee, Ministry of Health Malaysia (NMRR-22-00545-XAC). This secondary analysis was conducted using de-identified data released for research purposes, with approval from the Director General of Health Malaysia. All procedures adhered to the principles of the Declaration of Helsinki.

## Result

### Characteristics of participants

Table 1 summarises the demographic and health characteristics of the study population. The age distribution of the overall sample was relatively balanced; however, the majority of PWDs were older adults (63.9%). The sex distribution was nearly equal, with 46.1% males and 54.3% females. Most participants were Malay (58.3%) and married (66.8%). A large proportion belonged to the Bottom 40% (B40) income group (62.7%), had attained secondary education (63.7%), and resided in urban areas (76.4%). With respect to body mass index (BMI), most participants were either of normal weight (35.5%) or overweight (31.9%). Regarding comorbidities, hypertension (64.0%) and hypercholesterolemia (57.3%) were the most prevalent among PWDs.

The overall prevalence of disability in the study population was 8.2% (95% CI: 7.4–9.2; *N* = 1,207). Among the six functional domains assessed, the highest prevalence was reported for difficulty in seeing (11.2%), followed by walking (10.2%) and remembering/concentrating (6.9%). Difficulties with hearing (3.2%), communication (1.8%), and self-care (1.5%) were less frequently reported. The distribution of disability by type of difficulty is presented in Table 2.

### Disability and morbidity

The overall prevalence of comorbidity among PWDs was 73.9%, markedly higher than that observed in the general population (45.9%). Hypertension was the most common morbidity among PWDs (56.9%), followed by hypercholesterolemia (51.8%) and diabetes mellitus (33.3%). The distribution of morbidities among PWDs and non-PWDs is presented in Table 3.

### Factors associated with morbidity among PWD

Table 4 presents the factors associated with morbidity among PWDs. The risk of morbidity was significantly higher among older PWDs, individuals of Malay ethnicity, and those with lower educational attainment. Younger PWDs aged 18–35 years [aOR = 0.123; 95% CI: 0.052–0.300] and 36–59 years [aOR = 0.443; 95% CI: 0.247–0.739] had substantially lower odds of morbidity compared with older PWDs. Ethnicity was also a determinant, with individuals categorised as “Others” [aOR = 0.360; 95% CI: 0.111–1.166] exhibiting a lower risk compared with Malays. Education emerged as a protective factor: PWDs with primary [aOR = 0.761; 95% CI: 0.355–1.631], secondary [aOR = 0.608; 95% CI: 0.350–1.057], or tertiary education [aOR = 0.270; 95% CI: 0.104–0.586] had reduced odds of morbidity relative to those with no formal education.

**Table 1:** Baseline characteristics of participants (*N* = 10,842).

Variables	Total *n* (%) *N* = 10,842	PWDs *n* (%) *N* = 1207	Non-PWDs *n* (%) *N* = 9635
Age			
18–29	2108 (19.4)	63 (5.2)	2045 (21.2)
30–39	2054 (18.9)	69 (5.7)	1985 (20.6)
40–49	1905 (17.6)	123 (10.2)	1782 (18.5)
50–59	1802 (16.6)	181 (15.0)	1621 (16.8)
> 60	2973 (27.4)	771 (63.9)	2202 (22.9)
Sex			
Male	4995 (46.1)	544 (45.1)	4451 (46.2)
Female	5847 (54.3)	663 (54.9)	5184 (53.8)
Ethnicity			
Malay	6318 (58.3)	665 (55.1)	5653 (58.7)
Chinese	1685 (15.5)	224 (18.6)	1461(15.2)
Indian	697 (6.4)	83 (6.9)	614 (6.4)
Bumiputera Sabah	1043 (9.6)	100 (8.3)	943 (9.8)
Bumiputera Sarawak	412 (3.8)	100 (8.3)	312 3.2)
Others	687 (6.3)	35 (2.9)	652 (6.8)
Marital status			
Single	2244 (20.7)	119 (9.9)	2125 (22.1)
Married	7240 (66.8)	757 (62.7)	6483 (67.3)
Widowed/ Divorcee	1349 (12.4)	328 (27.2)	1021 (10.6)
Household income			
B40	6802 (62.7)	876 (72.6)	5926 (61.5)
M40	2742 (25.3)	243 (20.1)	2499 (25.9)
T20	1266 (11.7)	86 (7.1)	1180 (12.3)
Locality			
Urban	8278 (76.4)	830 (68.8)	7448 (77.3)
Rural	2564 (23.7)	377 (31.2)	2187 (22.7)
Education level			
No formal education	1091 (10.1)	326 (27.0)	765 (7.9)
Primary education	1434 (13.2)	275 (22.8)	1159 (12.0)
Secondary education	6910 (63.7)	546 (45.2)	6364 (66.1)
Tertiary education	1372 (12.7)	55 (4.6)	1317 (13.7)
Body Mass Index			
Underweight	470 (4.3)	48 (3.9)	422 (4.4)
Normal	3853 (35.5)	382 (31.65)	3471 (36.02)
Overweight	3462 (31.9)	336 (27.8)	3126 (32.4)
Obese	2342 (21.6)	245 (20.3)	2097 (21.8)
Diabetes mellitus			
Yes	2304 (21.3)	473 (39.2)	1831 (19.0)
No	8535 (78.7)	734 (60.8)	7801 (81.0)
Hypertension			
Yes	4042 (37.3)	772 (64.0)	3270 (34.0)
No	6796 (62.7)	435 (36.0)	6361 (66.0)
Hypercholestherolemia			
Yes	4348 (40.1)	692 (57.3)	3656 (37.9)
No	6490 (59.9)	515 (42.7)	5975 (62.1)

**Table 2:** Prevalence by type of difficulty.

Type of difficulty	Prevalence
Seeing	11.1 (9.91, 12.48)
Hearing	3.2 (2.80, 3.81)
Walking	10.2 (9.29, 11.26
Remembering/ Concentrating	6.9 (6.02, 7.82)
Self-care	1.5 (1.28, 1.80)
Communicating	1.8 (1.49, 2.16)

**Table 3:** Percentage of morbidities among persons with disability (*N* = 1207).

	PWDs (*N* = 1207)	Non-PWDs (*N* = 9635)
Any morbidity	73.9 (69.7,77.8)	45.9 (44.0, 47.9)
Diabetes mellitus	33.3 (29.2, 37.6)	14.0 (12.8, 15.3)
Hypertension	56.9 (52.9, 60.8)	26.7 (25.2, 28.3)
Hypercholesterolemia	51.8 (47.1, 56.4)	31.7 (30.0, 33.4)

**Table 4:** Factors associated with comorbidities among persons with disability.

Variables	Unadjusted odds ratio (95% CI)	Adjusted odds ratio (95% CI)
Age		
> 60	*R*	*R*
18–35	0.085 (0.050, 0.145) **	0.123 (0.051,0.300 **
36–59	0.369 (0.234, 0.581) **	0.443 (0.247, 0.793) **
Sex		
Female	*R*	*R*
Male	0.847 (0.56, 1.271)	0.847 (0.540, 1.328)
Ethnicity		
Malay	R	R
Chinese	0.793 (0.476, 1.321)	0.639 (0.325, 1.254)
Indian	1.610 (0.802, 3.231)	0.906 (0.407, 2.020)
Bumiputera Sabah	1.014 (0.459, 2.241)	0.618 (0.272, 1.406)
Bumiputera Sarawak	1.958 (1.084, 3.537)	1.918 (0.957, 3.842)
Others	0.585 (0.173, 1.981)	0.360 (0.111, 1.166) *
Marital status		
Widowed/ Divorcee	R	R
Single	0.135 (0.073, 0.250) **	0.604 (0.247, 1.477)
Married	0.479 (0.305, 0.754) **	0.896 (0.512, 1.571)
Household income		
B40	R	R
M40	0.548 (0.350, 0.858) *	0.825 (0.491, 1.388)
T20	0.527 (0.287, 0.930)	0.837 (0.455, 1.574)
Locality		
Urban	R	R
Rural	0.794 (0.524, 1.204)	0.668 (0.423, 1.055)
Education level		
No formal education	R	R
Primary education	0.802 (0.410, 1.569) **	0.761 (0.355, 1.631) *
Secondary education	0.474 (0.300, 0.750) **	0.608 (0.350, 1.057) *
Tertiary education	0.183 (0.0800.416) *	0.270 (0.104, 0.586) *

## Discussion

This study examined factors associated with morbidity among PWDs in Malaysia using nationally representative data. The prevalence of disability was 8.2%, reflecting a reduction compared with previous years^25^, yet still within the range reported internationally (1.8%–26.8%), depending on the measurement instruments^1,20–22^. Vision and mobility difficulty emerged as the most prevalent forms of disability, consistent with patterns reported by the Disability Data Initiative^30^. Importantly, more than two-thirds of PWDs experienced at least one morbidity, with hypertension being most common, rates that exceeded those reported in earlier studies^5,14^. Older age, marital status, and locality were reported to be significantly associated with morbidity among PWDs. Additionally, ethnic minorities and low levels of education were at higher risk of morbidity among PWDs.

Age emerged as a key determinant of morbidity among PWDs. Consistent with global findings, disability was most prevalent among older adults (26.0%), comparable to estimates of 20.3% – 39.2% in other settings^5,31^. The higher prevalence of morbidity in older adults likely reflects cumulative exposure to oxidative stress and chronic inflammation^32^, and lifestyle risk factors associated with ageing^33^. Additionally, ageing is a well-documented risk factor for disability^21,31,34^, probably due to age-related declines in muscle strength^35^. Given the likelihood of multimorbidity in this group, which often necessitates polypharmacy, older PWDs face an amplified vulnerability due to existing barriers in healthcare access. Although gender was not a statistically significant factor in this study, the higher prevalence of disability and morbidity among females, coupled with their well-documented barriers to healthcare access, warrants greater attention^36–39^. Female older adults, in particular, experience reduced quality of life^40^, underscoring the need for gender-sensitive interventions.

Socioeconomic disparities were strongly associated with morbidity among PWDs. Disability itself can restrict employment opportunities, thereby reducing socioeconomic status^1,2,22^. Low socioeconomic position has also been linked to greater morbidity risks, likely due to psychosocial stress and reduced capacity to adopt healthier behaviours^41,42^. Limited financial resources and healthcare costs further constrain access to care among PWDs^18,38,39,43^. Education level, as a proxy for socioeconomic status, was found to be protective: PWDs with higher education had significantly lower morbidity risks. This finding is consistent with evidence linking low education to higher risks of diabetes, hypertension^44^ and NCDs^45^. Additionally, research on disability has also highlighted low education levels as a significant risk factor^2,22^, possibly due to limited health literacy^46^, such as limited knowledge about disease prevention and management, as seen in studies on dietary sodium and related health conditions.

Interestingly, PWDs residing in rural areas had a lower risk of morbidity compared to their urban counterparts. This pattern aligns with evidence showing higher blood pressure, greater body fat, lower physical activity, and higher smoking prevalence among urban residents^47,45^. Such urban lifestyle factors may contribute to the observed disparities.

Ethnic differences were also apparent. PWDs from Bumiputera Sarawak groups experienced higher morbidity risks compared with Malays. These findings are consistent with previous reports from NHMS 2015, which demonstrated higher disability prevalence among ethnic minorities^25^. Possible explanations include disparities in healthcare awareness, access, and lifestyle practices. For example, prior research reported that Chinese and “other” ethnic groups in Malaysia were more likely to engage in healthier lifestyle behaviours^33^. These findings highlight the need for culturally tailored interventions to address ethnic disparities in morbidity among PWDs.

Marital status also influenced morbidity, with married and divorced PWDs experiencing higher risks. This may be partly attributable to age, as individuals in these categories are typically older. Prior research has similarly reported greater morbidity risks among married, separated, and divorced women^48,49^, as well as elevated disability risks among widowed or separated individuals^21^.

Together, these findings highlight that both morbidity and disability independently and jointly reduce quality of life and increase mortality. Two strategies are critical for addressing these challenges: (1) improving equitable access to healthcare services for PWDs, and (2) strengthening rehabilitation for individuals with NCDs to prevent progression to disability. Targeted preventive strategies are urgently needed for disadvantaged groups, including ethnic minorities, individuals with low education, older adults, and women. Health promotion should prioritise improving health literacy and lifestyle behaviours within these vulnerable populations. In addition, government initiatives should prioritise improving accessibility, including transportation services, while the Ministry of Health should strengthen capacity building by enhancing healthcare providers’ training in the management of PWDs^50,51^. Emerging approaches, such as precision and personalised medicine, may further enhance outcomes by tailoring management strategies to individual needs^52,53^.

This study has several strengths. It used data from a nationally representative survey, enhancing generalisability, and employed the Washington Group Short Set (WGSS), a globally recognised and validated tool for disability assessment. However, certain limitations should be acknowledged. The measurement of morbidity was restricted to diabetes, hypertension, and hypercholesterolemia, and did not capture the full spectrum of NCDs. Mental health conditions, which are closely linked to both disability and morbidity^54,55^, were also not assessed in this analysis. Future studies incorporating a broader range of physical and mental health conditions are warranted to provide a more comprehensive understanding of morbidity among PWDs.

## Conclusion

The prevalence of disability in this study is comparable to that of other countries, such as Beijing and Bangladesh. However, findings indicate that more than half of PWD have comorbidities, with half affected by hypertension and one in three diagnosed with diabetes. These results highlight the urgent need for targeted preventive strategies, particularly for older individuals, urban dwellers, ethnic minorities, and PWD with lower education levels, to mitigate the burden of morbidity among this vulnerable population.

## Data Availability

All data related to this study are included in the manuscript. However, the data are not publicly available. Access to the complete dataset can be obtained from the Institute for Public Health, Ministry of Health Malaysia, upon reasonable request and with permission from the Director-General of Health Malaysia.
